# Association between platelet-to-high-density lipoprotein cholesterol ratio and future stroke risk: a national cohort study based on CHARLS

**DOI:** 10.3389/fneur.2024.1479245

**Published:** 2024-11-13

**Authors:** Xin Hou, Meibao Zhu, Zhenghao Zhu, Yanhui Li, Xinmin Chen, Xiaohong Zhang

**Affiliations:** Department of Cardiology, The Third Affiliated Hospital of Anhui Medical University, Hefei, Anhui, China

**Keywords:** platelet-to-high-density lipoprotein cholesterol ratio, stroke, cohort study, CHARLS, high-density lipoprotein cholesterol

## Abstract

**Background:**

According to recent research, there is a considerable correlation between the severity of coronary artery disease and the platelet-to-high-density lipoprotein cholesterol ratio (PHR), which suggests that PHR is a potentially valuable inflammatory biomarker. However, the body of current research offers insufficiently strong evidence to clarify the connection between PHR and the incidence of stroke. Therefore, this study aims to elucidate any potential associations between PHR and stroke risk.

**Methods:**

This study employed data from the China Health and Retirement Longitudinal Study (CHARLS) covering the period from 2011 to 2018. It included 5,872 participants who did not have a history of stroke in 2011. These patients were separated into four groups according to their baseline PHR quartiles. The main goal of the study was to focus on stroke outcomes. Stroke was defined as an occurrence of a cerebrovascular accident confirmed by a physician. We employed Cox proportional hazards regression models to investigate the association between PHR and the likelihood of experiencing a stroke. Furthermore, we conducted restricted cubic spline regression analysis and subgroup analysis.

**Results:**

The average follow-up period was 77.5 months, during which 390 participants experienced a stroke. In comparison to the lowest quartile group, participants in the highest quartile of PHR had a 49% increased risk of stroke (HR 1.49, 95% CI 1.13–1.96, *p* = 0.004). The adjusted multivariable Cox regression analysis maintained the statistical significance of this association (aHR 1.42, 95% CI 1.06–1.90, *p* = 0.019). After adjustment, a positive linear relationship between stroke risk and PHR was identified through restricted cubic spline regression analysis (nonlinear *p* > 0.05). Additionally, the impact of stroke was consistent across a variety of subgroups, as evidenced by subgroup analysis.

**Conclusion:**

Our study indicates that higher PHR levels are significantly associated with an increased risk of stroke and that these levels can be used to identify groups that are at high risk of stroke.

## Introduction

Stroke is a localized neurological impairment lasting over 24 h or fatal (1). The Global Burden of Disease Study in 2019 found that stroke burden increased significantly from 1990 to 2019. Stroke incidence grew 70.0%, stroke-related fatalities 43.0%, and stroke-related DALYs 32%. In 2019, it was estimated that there were 12.2 million new stroke cases globally, with an estimated total of 101 million affected individuals. Stroke has emerged as the third leading cause of the global disease burden. With a total of 28.76 million stroke patients, there were 3.94 million new instances of stroke in China in 2019. This is a 124% increase since 1990. China is facing one of the most significant stroke challenges globally (2). Thus, identifying and managing stroke risk factors is critical for reducing this societal burden.

Atherosclerosis develops and progresses due in large part to inflammation, with platelets being essential to this process (3, 4). A growing amount of research indicates a strong correlation between high levels of inflammatory biomarkers and stroke (5–7). The development of atherosclerosis and related inflammation depends on platelet activation and aggregation (8). Additionally, there is an increasing acknowledgement of the critical function of platelets in immune responses and inflammation (9). Previous research has indicated that platelets, as a core component of inflammatory markers and the coagulation cascade, are important in assessing stroke risk (10).

In contrast, it has been demonstrated that high-density lipoprotein cholesterol (HDL-C) has a number of anti-thrombotic, anti-inflammatory, and anticoagulant properties (11–13). Research has indicated a potential correlation between an elevated risk of stroke and reduced levels of HDL-C. As a result, HDL-C and platelet count are important markers of inflammatory alterations and blood rheology.

The platelet-to-high-density lipoprotein cholesterol ratio (PHR) has recently emerged as a potential inflammatory biomarker. It is thought to be a good predictor of metabolic syndrome, non-alcoholic fatty liver disease, and liver fibrosis (14, 15). Not only that, but new studies have linked high PHR levels to poor long-term health outcomes in T2DM and CAD patients (16). Zhang et al. (17) found that an increase in PHR levels is associated with an increased risk of cardiovascular disease mortality in patients with depression. The study by Wang et al. (18) found a significant association between PHR levels and the prevalence of heart failure. The study conducted by Zhang et al. demonstrated a direct association between PHR and the occurrence of stroke, as well as a direct linear connection between PHR and the death rate from cardiovascular disease in individuals who had survived a stroke (19). Nevertheless, the existing study on the association between PHR and stroke is currently insufficient, and additional investigation into their connection is required. This study employed data from the China Health and Retirement Longitudinal Study (CHARLS) spanning from 2011 to 2018 to carry out a prospective cohort analysis. The aim is to investigate the correlation between PHR and the occurrence of new-onset stroke, in order to provide additional reference materials for stroke prevention in middle-aged and older adults in China.

## Methods

### Data source and study population

In this study, data from the China Health and Retirement Longitudinal Study (CHARLS) were used for a secondary analysis. A thorough nationwide longitudinal research called CHARLS^1^ was created to evaluate the population’s social, health, and economic circumstances (19). As previously reported in publications (19), the CHARLS cohort was created by means of a multi-stage probability sampling procedure that selected participants from 450 villages in 150 counties spread across 28 provinces. Target people 45 years of age and older were included in the baseline survey, which was finally completed by 12,115 participants between June 2011 and March 2012. During individual interviews, data were gathered using standardized questionnaires, and follow-up interviews were held around every 2 years. The Biomedical Ethics Review Committee of Peking University in China granted ethical approval for the CHARLS project (IRB00001052-11015). Prior to their involvement in the study, all research participants provided written consent. The CHARLS project website provides public access to the datasets that are pertinent to this investigation (19).

**Figure 1:**
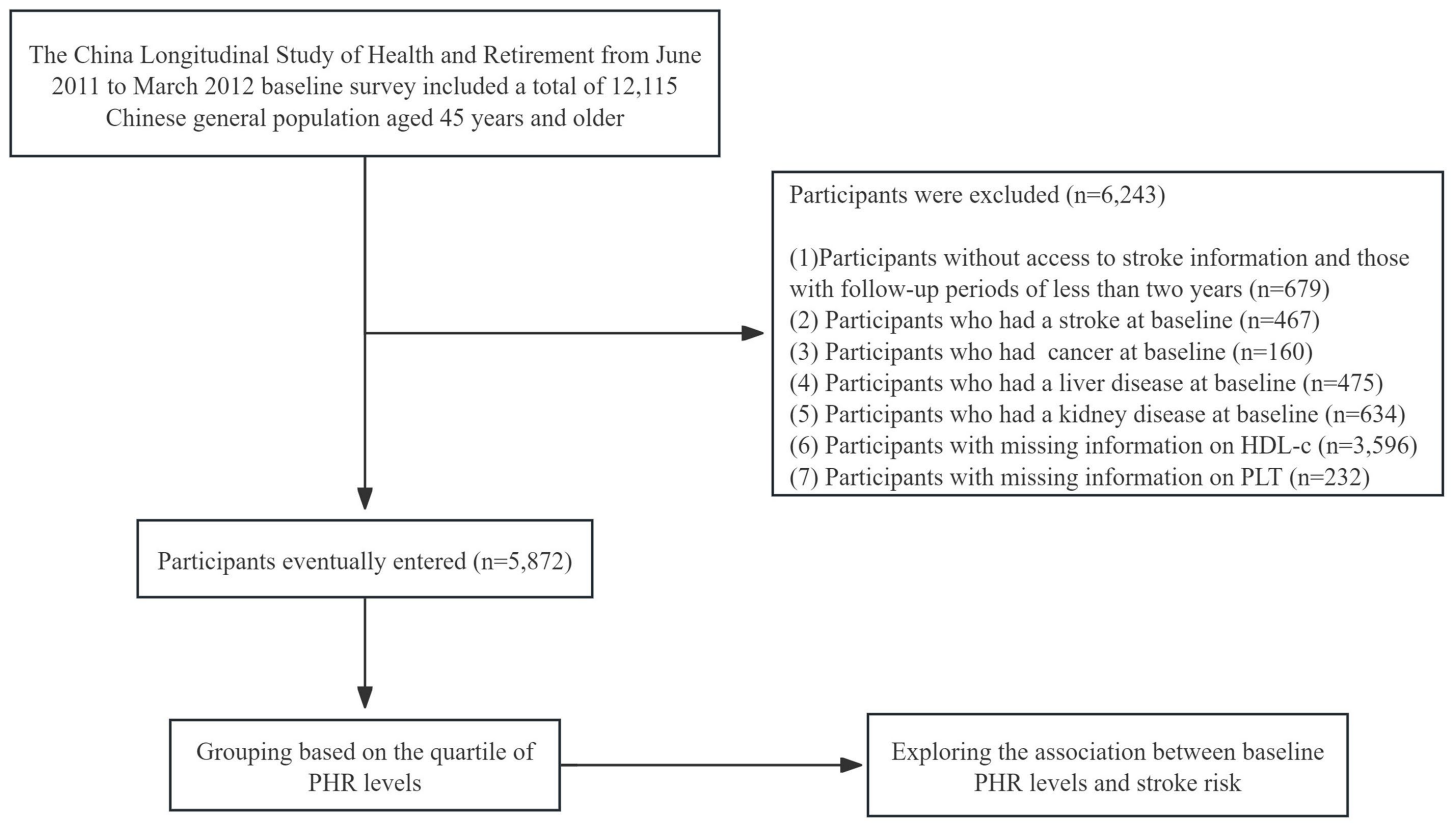
Flow chart of the study.

Our study used 2011–2018 CHARLS survey data. The baseline survey (2011–2012) was followed by three participant follow-ups (2013–2014, 2015–2016, 2017–2018). Participants without a history of stroke were primarily included in the current analysis. Individuals with baseline malignant tumors, liver diseases, and kidney diseases were excluded from the study. People who had not been followed up on for more than 2 years and people whose stroke data was not full were also taken out. People who did not have baseline platelet counts or HDL-C levels were also thrown out (Figure 1 shows a full overview of the recruitment process). This study included a total of 5,872 participants. Based on prior study experience (15), we separated the individuals into four subgroups based on their PHR quartiles.

### Data collection

Interviewers employing Computer-Assisted Personal Interviewing (CAPI) techniques were trained by Beijing University CHARLS staff to perform household surveys (20). Sections on demographics, functioning, health status, diagnosis of chronic diseases, and health-related habits including drinking and smoking as well as physical activity are all included in the basic CHARLS questionnaire. The interviewers were also capable of taking the participants’ blood pressure, height, and weight in order to gauge their physical health. Medical staff from the Chinese Center for Disease Control and Prevention took fasting venous blood samples in accordance with routine procedures, and the samples were then examined in the center’s laboratory.

### PHR

The calculation method for PHR is the plasma platelet count (10^9/L) divided by the plasma high-density lipoprotein cholesterol level (mmol/L).

### Stroke diagnosis

Individuals who did not have a previous stroke at the beginning of the study who later reported experiencing a stroke during the follow-up period were documented as new cases. Stroke incidence data were methodically gathered via questionnaires, which inquired about participants’ medical diagnosis of stroke, the date of diagnosis or awareness of the condition, and their utilization of stroke treatment. Positive answers given during subsequent inquiries were categorized as initial stroke diagnoses, with the date provided indicating the beginning of the condition. The timing of stroke occurrence was determined by calculating the interval between stroke onset and baseline measurement. The follow-up period for patients who did not report a stroke during follow-ups was determined by calculating the gap between the baseline evaluation and the last survey date (20).

### Covariates

Prior research and clinical experience informed the choice of variables (21, 22). The following are the variables that were considered: (i) categorical variables: gender, smoking status, drinking status, hypertension, diabetes mellitus (DM), chronic lung disease (CLD); (ii) continuous variables: age, body mass index (BMI), systolic blood pressure (SBP), diastolic blood pressure (DBP), C-reactive protein (CRP), hemoglobin concentration (HGB), platelet (PLT), fasting plasma glucose (FPG), hemoglobin A1c (HBA1c), serum triglyceride (TG), total cholesterol (TC), high-density lipoprotein cholesterol (HDL-C), low-density lipoprotein cholesterol (LDL-C), blood urea nitrogen (BUN), uric acid (UA), serum creatinine (Scr), cystatin C, triglyceride glucose index (TyG = ln[FPG (mg/dL) × TG (mg/dL) / 2]), TyG-BMI (BMI × TyG index).

### Missing data processing

This study experienced missing data, including hypertension (29, 0.49%), DM (38, 0.65%), CLD (6, 0.10%), smoking status (105, 1.79%), drinking status (2, 0.03%), BMI (893, 15.21%), SBP (896, 15.26%), DBP (896, 15.26%), CRP (14, 0.24%), HGB (2, 0.03%), FPG (1, 0.02%), HBA1c (58, 0.99%), TG (1, 0.02%), TC (1, 0.02%), LDL-C (12, 0.20%), BUN (1, 0.02%), UA (1, 0.02%), Scr (4, 0.07%), cystatin C (1,401, 23.86%), TyG index (1, 0.02%), and TyG-BMI (894, 15.22%). In order to mitigate the bias that can result from missing variables, which can impede the precise representation of the statistical power of the target sample during the modeling phase, multiple imputations were implemented in accordance with the missing data procedure described by White and Groenwald (23, 24). Incorporating the aforementioned missing variables into the estimation model, where SBP, DBP, HGB, FPG, HbA1c, TC, LDL-C, BUN, UA, Scr, cystatin C, and TyG conform to a normal distribution, and the regression type is linear regression. The remaining variables do not conform to a normal distribution, and the regression type is generalized linear model. The missing data analysis process uses the missing at random (MAR) assumption (23). The process of missing data analysis relied on the assumption of Missing At Random (MAR), which involved the creation of five imputed datasets and the subsequent combination of the results using the Markov Chain Monte Carlo method with chained equations.

### Statistical analysis

SPSS 26.0 and R 3.4.3 were used for statistical analysis. Two-sided *p*-values below 0.05 were considered significant. Baseline variables were compared between groups according to PHR quartiles. Categorical variables were reported using percentages and frequencies, whereas continuous variables were presented as median (interquartile range) or mean ± standard deviation (SD). Comparisons between PHR groups were made using χ2 for categorical variables and ANOVA or Kruskal-Wallis H for continuous variables.

Kaplan–Meier graphs showed stroke incidence rates by PHR quartile. PHR and stroke incidence were examined using univariate and multivariate Cox regression models. Hazard ratios and 95% confidence intervals (CI) were determined. This study used stepwise Cox regression analysis to choose clinically relevant factors for the multivariable model. Model 1 had no covariate adjustments; Model 2 had age (continuous) and gender adjustments; Model 3 had further adjustments for age, gender, smoking status, drinking status, hypertension, diabetes mellitus, chronic lung disease, CRP, HBA1c, TG, LDL-c, UA, Scr, cystatin C, and TyG-BMI. While controlling for the same covariates, restricted cubic splines (RCS) were employed to illustrate potential linear correlations between PHR and stroke risk. Based on prior literature and peer feedback (22), we performed subgroup analyses using a stratified Cox proportional hazards regression model for different subgroups (age, gender, hypertension, diabetes mellitus, smoking, drinking, TyG-BMI). In addition to stratification parameters, age, gender, hypertension, DM, CLD, smoking, drinking, BMI, CRP, FPG, HBA1C, TG, LDL-c, BUN, UA, Scr, cystatin C, and TyG-BMI were adjusted. The models with and without interaction terms were tested for interaction terms using likelihood ratio tests.

## Results

### Baseline characteristics of participants

A total of 5,872 participants were analyzed, with 2,700 males and 3,172 females completing follow-up visits. The average age of the participants was 58.76 ± 9.83 years. The mean value of the PHR was 82.64 ± 40.28, with a range of 3.49 to 446.97. The PHR followed a normal distribution. The basic data and biochemical characteristics of participants grouped by quartiles of PHR are shown in Table 1. The results indicate that parameters such as BMI, DBP, CRP, FPG, HBA1C, TG, TyG, and TyG-BMI all significantly increased with higher PHR values. In contrast, age, BUN, and Cystatin C displayed an opposite trend. Furthermore, the proportion of non-drinkers, females, individuals with hypertension, diabetes, and increased with higher PHR values, while the proportion of males decreased.

**Table 1:** The baseline characteristics of participants.

PHR quartile	Quartile 1 (<119.19)	Quartile 2 (119.19–162.91)	Quartile 3 (162.91–217.70)	Quartile 4 (≥217.70)	*p*
Participants (*n*)	1,468	1,468	1,468	1,468	
Age, Mean ± SD	60.35 ± 10.04	58.91 ± 9.80	57.89 ± 9.70	57.90 ± 9.57	<0.001
Gender, *n* (%)					0.016
Female	750 (51.09)	778 (53.00)	826 (56.27)	818 (55.72)	
Male	718 (48.91)	690 (47.00)	642 (43.73)	650 (44.28)	
Smoking status, *n* (%)					0.799
Never smoker	876 (59.67)	899 (61.24)	908 (61.85)	884 (60.22)	
Ever smoker	128 (8.72)	109 (7.43)	111 (7.56)	122 (8.31)	
Current smoker	464 (31.61)	460 (31.34)	449 (30.59)	462 (31.47)	
Drinking status, *n* (%)					<0.001
Never drinker	795 (54.16)	835 (56.88)	896 (61.04)	943 (64.24)	
Ever drinker	138 (9.40)	107 (7.29)	98 (6.68)	120 (8.17)	
Current drinker	535 (36.44)	526 (35.83)	474 (32.29)	405 (27.59)	
Hypertension, *n* (%)	327 (22.28)	294 (20.03)	405 (27.59)	451 (30.72)	<0.001
DM, *n* (%)	60 (4.09)	64 (4.36)	83 (5.65)	106 (7.22)	<0.001
CLD, *n* (%)	173 (11.78)	124 (8.45)	131 (8.92)	128 (8.72)	0.006
BMI [kg/m^2^, M (Q₁, Q₃)]	22.39 (20.38, 24.79)	22.83 (20.61, 24.94)	23.50 (21.37, 26.06)	23.91 (21.46, 26.70)	<0.001
SBP [mmHg, Mean ± SD]	127.35 ± 21.30	127.21 ± 21.34	127.88 ± 20.65	130.59 ± 22.06	<0.001
DBP [mmHg, Mean ± SD]	73.40 ± 12.12	73.74 ± 11.83	75.07 ± 12.30	76.57 ± 12.61	<0.001
CRP [mg/L, M (Q₁, Q₃)]	0.86 (0.47, 1.75)	0.89 (0.49, 1.96)	1.07 (0.58, 2.12)	1.30 (0.67, 2.70)	<0.001
HGB [g/L, Mean ± SD]	14.34 ± 2.51	14.22 ± 1.96	14.29 ± 2.01	14.21 ± 2.06	0.311
PLT [10^9^/L, Mean ± SD]	142.83 ± 44.74	196.77 ± 43.37	231.70 ± 47.05	282.37 ± 76.14	<0.001
FPG [mg/dL, Mean ± SD]	105.57 ± 29.98	107.66 ± 34.93	108.21 ± 32.29	113.83 ± 40.01	<0.001
HBA1c [%, Mean ± SD]	5.16 ± 0.64	5.22 ± 0.80	5.27 ± 0.81	5.30 ± 0.90	<0.001
TG [mg/dL, M (Q₁, Q₃)]	85.85 (65.49, 117.71)	95.58 (69.92, 133.63)	111.51 (81.42, 158.41)	142.49 (102.44, 215.94)	<0.001
TC [mg/dL, Mean ± SD]	193.10 ± 36.92	193.46 ± 37.74	196.58 ± 38.90	191.49 ± 42.37	0.004
HDL-C [mmol/l, Mean ± SD]	1.63 ± 0.40	1.40 ± 0.31	1.24 ± 0.25	0.99 ± 0.24	<0.001
LDL-C [mg/dL, Mean ± SD]	112.22 ± 32.16	117.62 ± 33.89	122.11 ± 34.57	112.73 ± 39.94	<0.001
BUN [mg/dL, Mean ± SD]	16.72 ± 4.85	16.15 ± 4.79	15.59 ± 4.26	15.03 ± 4.29	<0.001
UA [mg/dL, Mean ± SD]	4.45 ± 1.25	4.40 ± 1.21	4.43 ± 1.22	4.55 ± 1.34	0.007
Scr [mg/dL, Mean ± SD]	0.79 ± 0.18	0.78 ± 0.18	0.77 ± 0.18	0.77 ± 0.19	0.074
Cystatin C [mg/L, Mean ± SD]	1.05 ± 0.27	1.00 ± 0.24	0.98 ± 0.24	0.96 ± 0.25	<0.001
TyG, Mean ± SD	8.43 ± 0.55	8.55 ± 0.58	8.72 ± 0.60	9.03 ± 0.75	<0.001
TyG-BMI, M (Q₁, Q₃)	188.63 (169.34, 211.00)	193.88 (173.90, 216.66)	205.33 (181.82, 229.39)	215.88 (190.26, 243.45)	<0.001

SD, standard deviation; DM diabetes mellitus; CLD, chronic lung disease; BMI, body mass index; SBP, systolic blood pressure; DBP, diastolic blood pressure; CRP, c-reactive protein; HGB, hemoglobin concentration; PLT, platelet; FPG, fasting plasma glucose; HBA1c, hemoglobin A1c; TG, triglyceride; TC, total cholesterol; HDL-C, high-density lipoprotein cholesterol; LDL-C, low-density lipoproteins cholesterol; BUN, blood urea nitrogen; UA, uric acid; Scr, serum creatinine; TyG, triglyceride glucose index; TyG-BMI, triglyceride glucose-body mass index.

### Relationship between PHR and stroke risk

**Figure 2:**
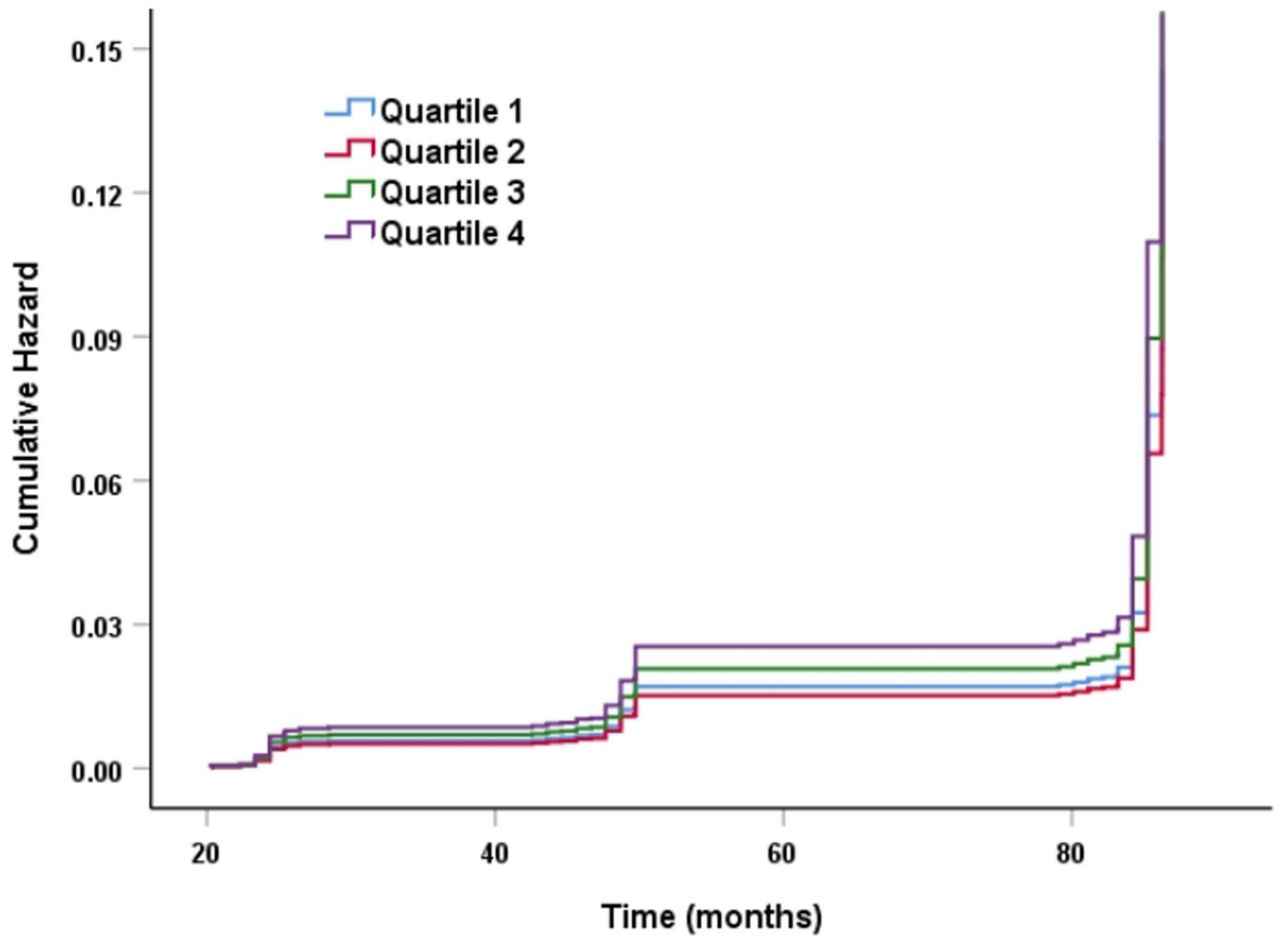
K–M plot of stroke by PHR level.

**Figure 3:**
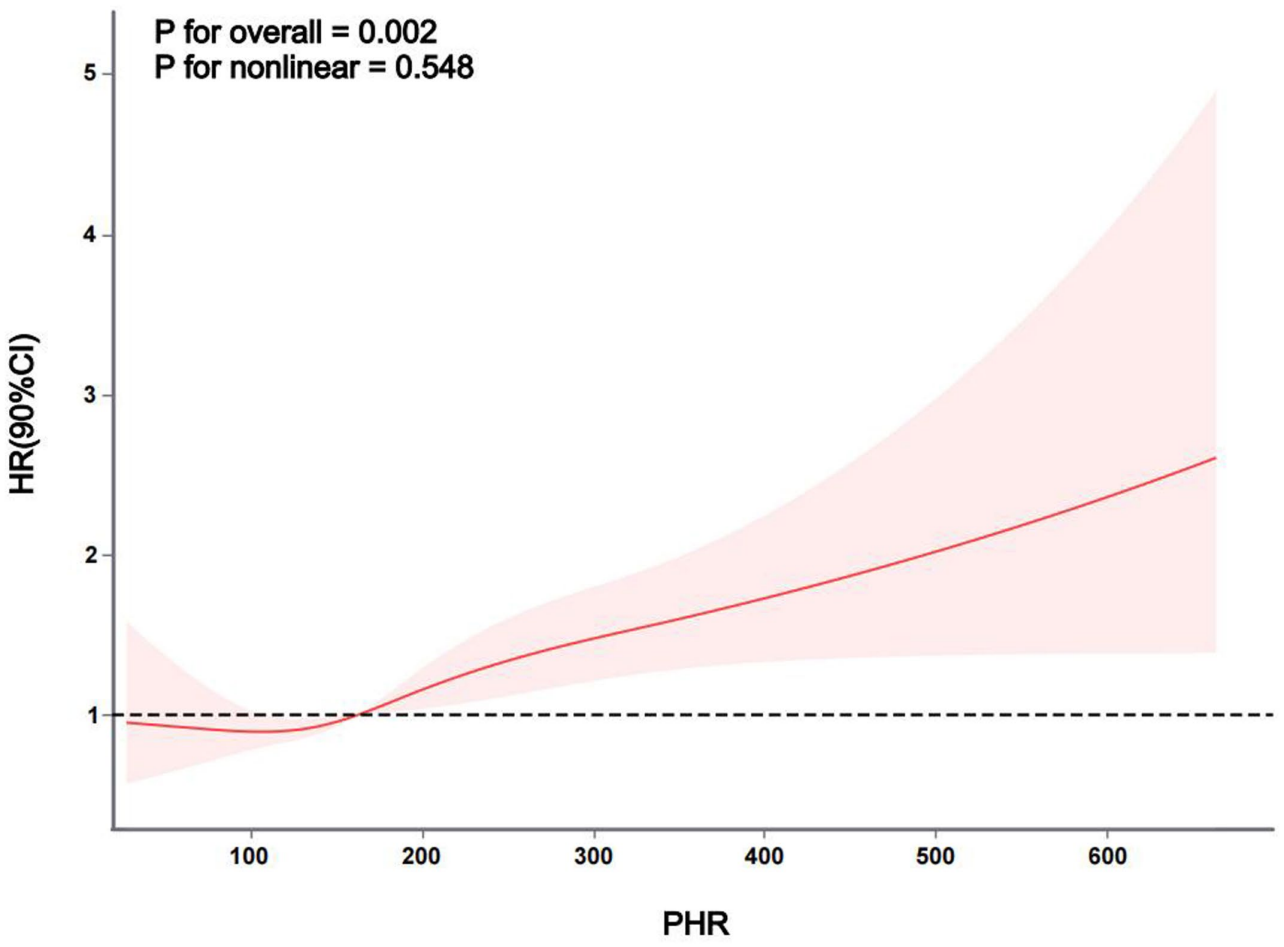
The association between PHR and stroke risk. The model was adjusted for age, gender, smoking, and drinking status, hypertension, diabetes mellitus, chronic lung disease, CRP, HBA1c, TG, LDL-c, UA, Scr, cystatin C, and TyG-BMI.

The Kaplan–Meier curve of cumulative stroke incidence among all study participants is depicted in Figure 2. Three Cox proportional hazards regression models were constructed to investigate the correlation between stroke risk and PHR, as illustrated in Table 2. In Model 1, the risk of stroke was 49% higher for participants in the highest quartile of PHR than for those in the lowest quartile (HR 1.49, 95% CI 1.13–1.96, *p* = 0.004). Age and gender were adjusted in Model 2. Adjustments were implemented in Model 3, which included hypertension, diabetes, chronic lung disease, CRP, HBA1c, TG, LDL-C, UA, Scr, Cystatin C, and TyG-BMI, as well as age, gender, smoking, and drinking status. The results of the multivariable Cox regression analysis were statistically significant (adjusted HR 1.42, 95% CI 1.06–1.90, *p* = 0.019). Additionally, restricted cubic spline analysis demonstrated a linear correlation between stroke risk and PHR after taking into account the aforementioned factors (Figure 3, *p* for non-linearity >0.05). The risk of stroke rises by 2% for every 10 SD increase in PHR (HR: 1.002, 95% CI 1.001–1.003); there is no discernible threshold or saturation relationship between the two.

**Table 2:** Relationship between PHR and the risk of stroke in different models.

PHR	Events/Subjects 390/5872	Model 1 HR (95%CI)	*p*	Model 2 HR (95%CI)	*p*	Model 3 HR (95%CI)	*p*
Quartile 1	88/1468	1.00 (Reference)		1.00 (Reference)		1.00 (Reference)	
Quartile 2	75/1468	0.89 (0.65–1.21)	0.464	0.95 (0.69–1.29)	0.722	0.93 (0.68–1.27)	0.641
Quartile 3	103/1468	1.22 (0.92–1.62)	0.175	1.29 (0.96–1.71)	0.087	1.23 (0.92–1.65)	0.159
Quartile 4	124/1468	1.49 (1.13–1.96)	0.004	1.49 (1.13–1.97)	0.005	1.42 (1.06–1.90)	0.019

Model 1: Not adjusted.

Model 2: Adjusted for age and gender.

Model 3: Adjusted for age, gender, smoking, and drinking status, hypertension, diabetes mellitus, chronic lung disease, CRP, HBA1c, TG, LDL-c, UA, Scr, cystatin C, and TyG-BMI.

### Subgroup analysis

**Figure 4:**
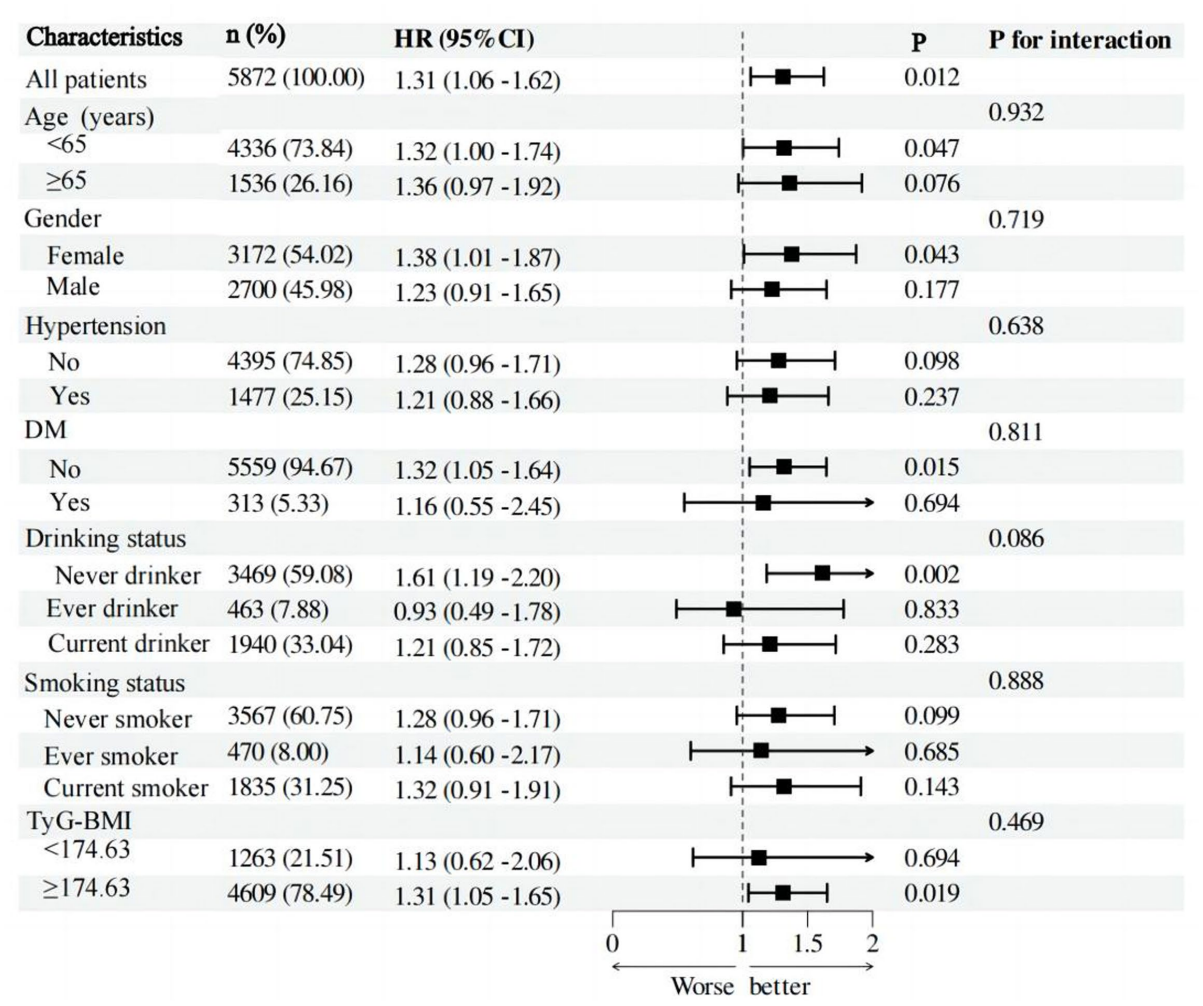
Forest plot of stroke risk according to different subgroups. The analysis with adjustment for age, gender, hypertension, DM, CLD, smoking status, and drinking status, BMI, CRP, FPG, HBA1C, TG, LDL-c, BUN, UA, Scr, cystatin C, and TyG-BMI. In each case, the model is not adjusted for the stratification variable.

Additional examination of post-hoc subgroup data reveals that the association between PHR and the risk of stroke is not affected by factors such as age, gender, hypertension, diabetes, alcohol consumption, smoking status, and TyG-BMI (Figure 4). Put simply, there is no statistically significant relationship between these variables and PHR (*p* > 0.05 for interaction).

## Discussion

To our knowledge, this is the first cohort study to investigate the association between PHR and stroke risk in China’s middle-aged and older population. The results of this study demonstrated a strong correlation between raised PHR and an increased risk of stroke. According to the subgroup analysis and interaction test, this connection was consistent across diverse demographic settings. Attention to PHR in clinical practice may help assess the risk of stroke in middle-aged and older adults. It provides additional references to facilitate clinical consultations and optimize stroke prevention decisions.

Jialal et al. first suggested the platelet-to-high-density lipoprotein cholesterol ratio (PHR) as a useful biomarker for predicting metabolic syndrome (MetS). Research indicates that elevated PHR significantly increases the risk of developing type 2 diabetes in obese individuals (25). Wu et al. (16) discovered that among diabetic patients, those with higher PHR levels demonstrated a statistically significant increase in all-cause mortality and cardiac mortality. Additionally, after correcting for potential confounders, PHR demonstrated a positive linear correlation with both overall and cardiac mortality. When PHR was less than 223.684 (OR: 0.97, 95% CI: 0.87–1.08, *p* = 0.53), Zhang et al.’s study found no significant correlation between PHR and stroke; however, when PHR was greater than 223.684, there was a significant increase in the odds of stroke (19). This result is generally in line with our study, which showed that individuals in the highest PHR quartile had a considerably higher risk of stroke than those in the lowest quartile. Even after accounting for a number of covariates, this connection was still statistically significant in the multivariable Cox regression analysis (aHR 1.42, 95% CI 1.06–1.90, *p* = 0.019). In contrast, there was no discernible correlation found in the second or third quartiles between PHR and stroke. Furthermore, our study found a linear link between PHR and the risk of stroke, in contrast with previous studies that suggested a non-linear relationship between PHR and stroke. The discrepancy may be attributed to several factors. Firstly, the difference in study type; the previous research was a cross-sectional study, whereas our study was an observational cohort study. Secondly, the inconsistency in the study population; the previous research focused on the general population, while our study specifically targeted individuals aged >45 years. Furthermore, due to methodological differences, there were variations in the adjusted covariates. Notably, we excluded participants with cancer, liver diseases, and kidney diseases at baseline due to the possible impact of these conditions on platelets and HDL cholesterol.

PHR exhibits exceptional sensitivity and specificity in the prediction of cardiovascular risk as a potential serum biomarker. Some previous studies have investigated the relationship between PHR and diabetes, inflammatory responses, and cardiovascular diseases, reflecting that PHR may represent an inflammatory and prothrombotic state (25–27). Previous research has demonstrated that elevated PHR levels are linked to multiple coronary artery stenoses and are correlated with the severity of CAD as measured by the Gensini score, indicating a connection between PHR and coronary artery atherosclerosis (28). The relationship between stroke and PHR can be elucidated through the lenses of inflammation and thrombus formation. Systemic inflammation is a critical factor in the development of stroke (6). Atherosclerosis, a significant pathogenic mechanism for ischemic and hemorrhagic strokes, is initiated and progressed by inflammation (29–31). HDL-C possesses anti-inflammatory properties, whereas platelets are critical markers for the assessment of systemic inflammation (12, 32). The relationship between platelets and inflammation in the progression of atherosclerosis has been previously elucidated in numerous studies (33–35). The recruitment of immune cells to plaques is facilitated by factors released by platelets, which creates a pro-inflammatory environment that is conducive to the development of atherosclerosis (36). According to a recent investigation, the risk of cardiovascular events may be elevated by the pathological state of platelets in T2DM (37). Decreased HDL-C levels impede the effective transport of cholesterol, resulting in cholesterol deposition within arterial walls and the formation of atheroma plaques (38). The primary protein in extracellular fluids, HDL-C, is essential for a variety of physiological functions, including inhibiting platelet activation and adhesion, functioning as an anti-inflammatory lipid, and facilitating cholesterol efflux (39, 40). An association between adverse cardiovascular outcomes and decreased serum HDL levels has been consistently demonstrated in observational studies and meta-analyses (41, 42).

There is a substantial correlation between elevated PHR and elevated levels of triglycerides and clotting factors, which implies that elevated PHR may indicate an imbalance in coagulation status. Patients who have experienced an ischemic stroke frequently exhibit coagulation abnormalities and post-stroke complications are frequently associated with coagulation dysfunction (43). Mechanistically, the direct risk associated with elevated platelets is thrombus formation, as elevated platelets enhance blood clotting, contributing to the pathogenic mechanisms of inflammation, and thrombosis, and actively participating in the development of atherosclerosis (44, 45). In contrast, HDL-C has antithrombotic and antiplatelet characteristics (14, 15). The risk of thrombosis and HDL-C levels have been found to be negatively correlated in multiple epidemiological studies. It has been demonstrated that HDL-C is a reliable indicator of platelet-dependent thrombus development (46). Apart from its involvement in the reverse transport of cholesterol, HDL-C also has interactions with platelets, the coagulation cascade, and the function of endothelium (12). By preventing intraplatelet cholesterol overload and by binding to platelet high-density lipoprotein receptors such as apoER2 and scavenger receptor class B type I (SR-BI), native high-density lipoprotein reduces platelet hyperreactivity (47, 48). Natural high-density lipoprotein’s anti-thrombotic qualities are also linked to preventing the coagulation cascade and encouraging the breakdown of fibrin clots (49). Moreover, high-density lipoprotein promotes the synthesis of prostacyclin and nitric oxide by endothelial cells, both of which are powerful inhibitors of platelet activation. As a result, high-density lipoprotein has a complex anti-thrombotic action (50, 51).

There are some significant limitations to the study. First, it’s unclear whether the results apply to younger populations or other ethnic groups given that the demographic focus is on middle-aged and elderly Chinese people. Secondly, the self-reported nature of stroke history in this study makes it prone to inaccuracies. However, it has been shown that there is a high level of consistency between self-reported disease diagnoses and medical records regarding cardiovascular events (52). Third, the CHARLS survey’s stroke subtypes are indistinguishable; thus, this study covers the overall stroke condition and cannot be extrapolated to individual subtypes. Fourth, even after adjusting for recognized potential confounders, there may still be unmeasured or uncontrolled confounders, such as the lack of information on the history of lipid-lowering and antiplatelet medication use in the original data, which may have some potential impact on the results and conclusion of this study. Lastly, this observational study can only show a correlation between PHR and stroke risk; it is not able to demonstrate a causative relationship.

## Conclusion

This study discovered a strong correlation between middle-aged and older Chinese people’s raised PHR levels and their increased risk of stroke. PHR levels can be a useful tool for determining populations at high risk of stroke, which can help to optimize stroke prevention decisions and facilitate clinical consultations.

## Data Availability

Publicly available datasets were analyzed in this study. This data can be found at: http://www.isss.pku.edu.cn/cfps/.

## Footnotes

^1^http://charls.pku.edu.cn
